# Homebound Older Adults: Neighborhood Social Cohesion and Physical Disorder and Social Network Size

**DOI:** 10.1093/geroni/igaf122.3286

**Published:** 2025-12-31

**Authors:** Brian Fons, Kelly Vences, Angelina Gutierrez, Namkee Choi

**Affiliations:** The University of Texas at Austin, Austin, Texas, United States; The University of Texas School of Social Work, Austin, Texas, United States; The University of Texas School of Social Work, Austin, Texas, United States; The University of Texas at Austin, Austin, Texas, United States

## Abstract

Research has shown significant associations between neighborhood characteristics and health in older adults, but few focused on homebound older adults. We used the 2022-2023 U.S. National Health and Aging Trends to examine the associations of (1) homebound state (i.e., never/rarely going outside in the past month) with neighborhood social cohesion (NSC), neighborhood physical disorder (NPD), and social network size (N = 5,593); and (2) changes in the homebound state with the changes in NSC, NPD, and social network size (N = 4,907). Older adults reported their perceived NSC and the number of people in their social network. Interviewers rated NPD based on their observations. Logistic regression models were used for multivariable analyses. The results show that homebound older adults were 4.9% of the Medicare beneficiaries age 65+ years in 2022. The odds of a homebound state were lower among those with higher perceived NSC (aOR=0.83, 95% CI = 0.77-0.90). Of those who were interviewed in both 2022 and 2023, 154 (34.1%) were homebound in 2022 but not in 2023, 169 (33.0%) were not homebound in 2022 but homebound in 2023, and 162 (32.9%) were homebound in both years. Compared to those who continued to be or were newly homebound, those who were no longer homebound in 2023 had increased social network size (aOR=2.21, 95% CI = 1.24-3.94). Social care policies and programs for the increasing number of homebound older adults need to be both individual- and community-focused on improving their social capital, including fostering NSC and social connectedness among community residents.

